# Complex staged emplacement of a basaltic lava: The example of the July 1974 flow of Kīlauea

**DOI:** 10.1007/s00445-025-01817-0

**Published:** 2025-03-31

**Authors:** S. Biass, B. F. Houghton, E. W. Llewellin, K. C. Curran, T. Thordarson, T. R. Orr, C. E. Parcheta, P. Mouginis-Mark

**Affiliations:** 1 Department of Earth Sciences, University of Hawai‘I at Mānoa, Honolulu, HI 96822 USA; 2 Department of Earth Sciences, University of Geneva, CH-1205 Geneva, Switzerland; 3 Department of Earth Sciences, Science Labs, Durham University, Durham, DH1 3LE UK; 4 Faculty of Earth Sciences, University of Iceland, 101 Reykjavík, Iceland; 5 U.S. Geological Survey, Alaska Volcano Observatory, Anchorage, AK 99508 USA; 6 U.S. Geological Survey, Hawaiian Volcano Observatory, Hawaii Volcanoes National Park, Honolulu, HI 96718 USA; 7 University of Alaska Fairbanks, Alaska Earthquake Center, Fairbanks, AK 99775 USA; 8 Hawai‘I Institute of Geophysics and Planetology, University of Hawai‘I at Mānoa, Honolulu, HI 96822 USA

**Keywords:** Physical volcanology, Kīlauea, ʻAʻā and Pāhoehoe, Lava Drainage and Ponding, Fissure eruptions, Eruption dynamics

## Abstract

Basaltic lava flows can be highly destructive. Forecasting the future path and/or behavior of an active lava flow is challenging because topography is often poorly constrained and lava has a complex rheology and emplacement history. Preserved lavas are an important source of information which, combined with observations of active flows, underpins conceptual models of lava flow emplacement. However, the value of preserved lavas is limited because pre-eruptive topography and, thus, syn-eruptive lava flow geometry are usually not known. Here, we use tree-mold data to constrain pre-eruptive topography and syn-eruptive lava flow geometry of the July 1974 flow of Kīlauea (USA). Tree molds, which are formed after advancing lava encloses standing trees, preserve the lava inundation height and the final preserved thickness of lava. We used data from 282 tree molds to reconstruct the temporal and spatial evolution of the ~ 2.1 km-long July 1974 flow. The tree mold dataset yields a detailed dynamic picture of staged emplacement, separated by intervals of ponding. In some ponded areas, flow depth during emplacement (~ 5 m) was twice the preserved thickness of the final lava (2–3 m). Drainage of the ponds led to episodic surges in flow advancement, decoupled from fluctuations in vent discharge rate. We infer that the final breakout occurred after the cessation of fountaining. Such complex emplacement histories may be common for pāhoehoe lavas at Kīlauea and elsewhere in situations where the terrain is of variable slope, and/or where lava is temporarily perched and stored.

**Supplementary Information:**

The online version contains supplementary material available at 10.1007/s00445-025-01817-0.

## Introduction

Fluid, far-reaching lava flows are a destructive hazard at highly active basaltic volcanoes, such as Mount Etna (Sicily), Piton de la Fournaise (Reunion), Nyiragongo (Congo), Mount Cameroon (West Africa), Mauna Loa and Kīlauea (Hawai‘i). All of these areas have been exposed to lava hazards in recent times. Kīlauea has experienced two recent volcanic crises (in 2014–2015 and 2018) in which the principal eruptive hazard was entry of basaltic lava flows into vulnerable areas (Poland et al. 2016; Neal et al. 2019). The 2018 eruption alone destroyed over 700 buildings and displaced some 3,000 residents, at a total cost approaching $US 1 billion (Neal et al. 2019). Uncertainties associated with flow path, advance rate and timing, and flow stagnation, were major issues in 2018 and in the 2014–2015 crisis (Poland et al. 2016).

Improved understanding of lava flow emplacement processes is required to reduce uncertainty in hazard-relevant parameters during lava flow crises and, hence, to mitigate risk. This understanding requires knowledge of both the intrinsic properties of the lava and the extrinsic factors related to the environment that it flows through (Macdonald 1953; Hon et al. 1994; Gregg and Fink 2000; Dietterich et al. 2015; Harris and Rowland 2015; Biass et al. 2019). Detailed monitoring of active lava flows can yield valuable data on the intrinsic properties (rheology, crystal and vesicle contents, etc.) and extrinsic factors (e.g., through responsive LiDAR or photogrammetric reconstruction of the ground surface ahead of an advancing flow as well as the new lava as it is emplaced). The 2018 Kīlauea eruption has proven to be an exemplar in this regard (e.g., Dietterich et al. 2021; deGraffenried et al. 2021), yet such intensive monitoring is very rare because it is costly, labour intensive, and can be hazardous. Consequently, much of our understanding of lava flow emplacement comes from studies of preserved lavas from historical or ancient eruptions (Rowland and Walker 1987; Walker 1991; Calvari and Pinkerton 1998; Solana 2012; Orr et al. 2015; Dietterich et al. 2018; Latutrie et al. 2023; Biass et al. 2024a, b).

**Fig. 1:**
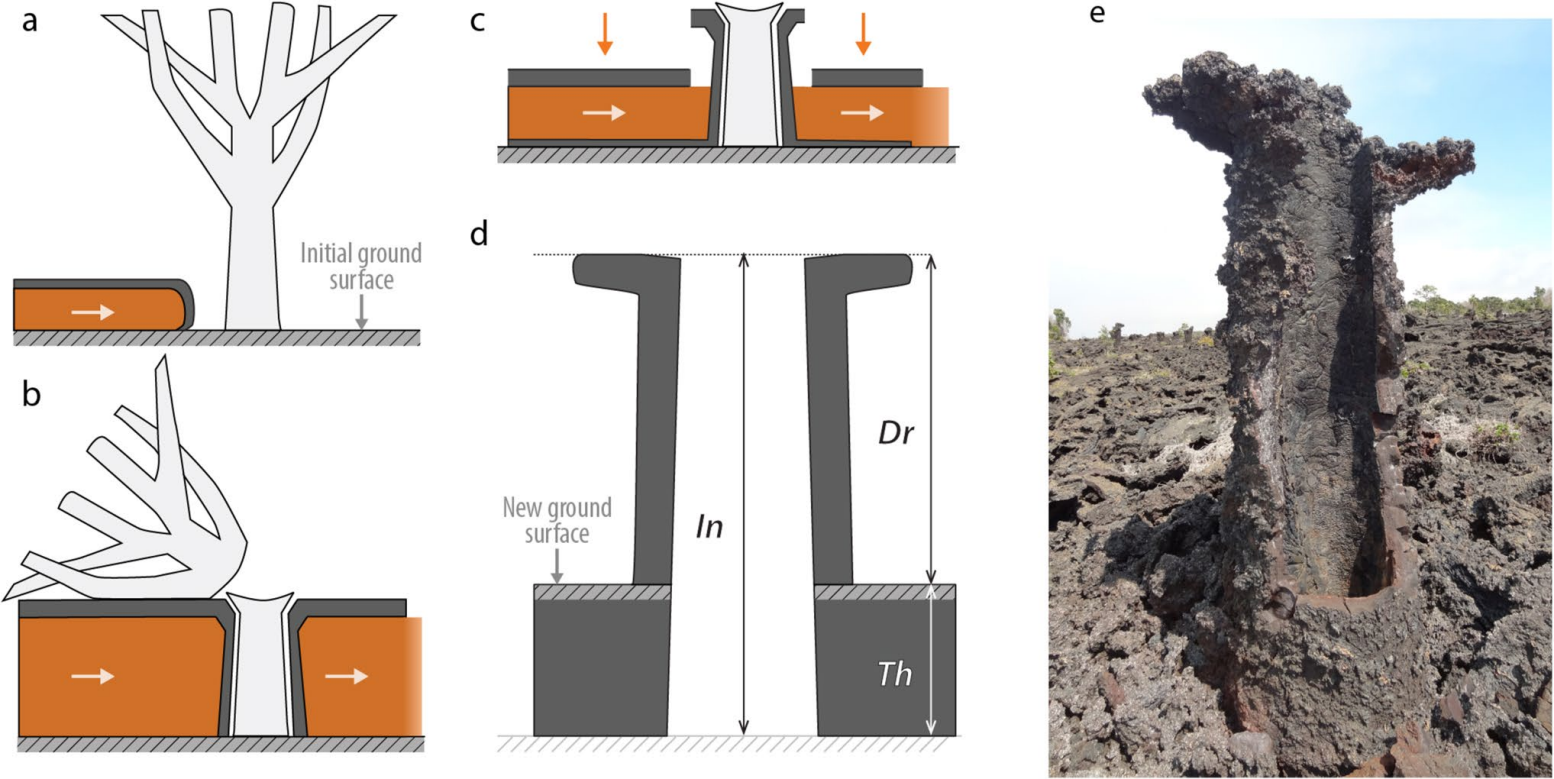
Conceptual model of the multi-stage emplacement of lava flow through time as preserved by the record of tree molds (**a-c**). **a.** The lava flow first spreads through the forest. **b.** It coats the tree, producing a quenched mold around the trunk that records the maximum inundation (*In*). The tree gradually burns through and falls onto the upper surface of the flow. **c.** If drainage occurs, the liquid core of the flow is remobilized down-flow and the flow surface drops to reach the final thickness (*Th*). **d.** Tree mold measurements in the field. The maximum level of inundation (*In*) and the drainage (*Dr*) are measured, from which the final flow thickness (*Th*) is calculated. e. Photograph of a ~ 1.5 m-tall tree mold on the July 1974 lava flow at Kīlauea. Note i) the pronounced collar left by the remnants of the crust formed when the flow stood at the maximum inundation height and ii) the higher elevation of the up-flow crust (left) caused by a bow-wave effect. Inundation heights were always recorded on the lee side

Studies of preserved lavas are of use in reconstructing both intrinsic properties and extrinsic factors – here we focus on extrinsic factors. Most old lavas generally offer only snapshots of its final upper surface and only partial information about: (1) the maximum height reached by the flow at peak thickness (i.e., the inundation height); (2) the extent of subsequent deflation by gas-escape or drainage; (3) final thickness; and (4) pre-eruption topography. Under favorable circumstances, lava tree molds, which form when lava quenches around trees (Fig. 1), preserve critical information that allows all four of these quantities to be reconstructed (Kosinski et al. 2012; Jones et al. 2017). Tree molds have previously been used to infer the chronology and dynamics of flow emplacement (e.g., Moore and Richter 1962; Lockwood and Williams 1978). If the level of the lava subsequently drops, the tree mold will then remain as a positive “lava-tree” above the final flow surface (Finch 1931; Lockwood and Williams 1978; Chevrel et al. 2019).

We thus undertook systematic mapping and characterization of an exceptionally well-preserved field of tree molds formed by the July 1974 eruption of Kīlauea. The data reveal a detailed picture of the spatio-temporal evolution of the flow field, involving complex and staged emplacement that was not described in contemporary reports. Since the dataset comprises both tree molds and lava-trees, we adopt the generic nomenclature of “tree molds” to describe both.

**Fig. 2:**
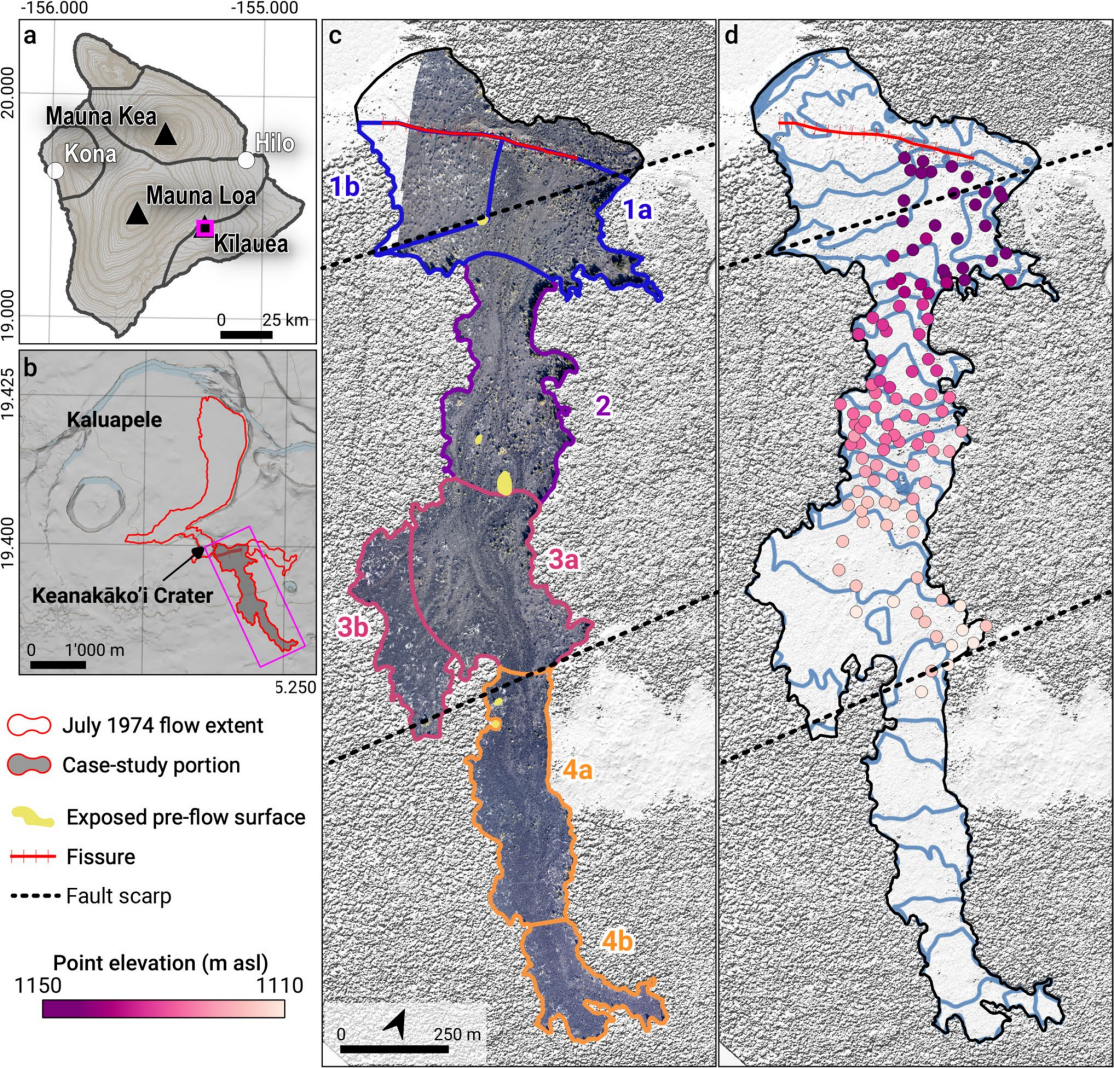
**a.** Location map for the study area on the Island of Hawaiʻi. Small pink rectangle outlines the area shown in Fig. 2b. **b.** Setting of the July 1974 lava flow, close to the southern margin of Kaluapele (Kīlauea’s summit caldera). Note that the topography reflects pre-March 2018 situation. The 1974 flows are outlined in red. In grey is the southern arm of July 1974 flow that is studied here. Pink rectangle outlines the area shown in Figs. 2c and d. **c.** Flow regions and sub-regions (colored numbers) defined from the geometry of the flow and its surface textures. Eruptive fissure is marked with red cross-hatching. The pre-eruptive surface within the area of the flow is only visible at five places (kīpuka), shown in yellow. Dashed black lines are pre-1974 faults mapped by Peterson (1967). The southeast corner of Keanakāko’i Crater lies directly under the letter c. The flow background is a 20-cm resolution orthophoto mosaic. **d.** Elevation of the height of maximum inundation by the July 1974 lava flow derived by two independent techniques. **1.** Blue lines are 2-m spaced smoothed elevation contours computed from the elevation model of Mouginis-Mark and Garbeil (2005) and indicate current slope. **2.** Colored dots show the absolute elevation (m above sea level) of the tops of a subset of intact tree molds that preserve the inundation height, from kinematic GPS measurements. The inundation height is relatively constant in sub-regions 1a and 3a but falls consistently to the south steeply in regions 2 and 4. High resolution versions of the orthophoto and hillshade of the DEM of Mouginis-Mark and Garbeil (2005) are provided in Online Resource 1. Background in **a** and **b** is from OpenStreetMap ( © OpenStreetMap contributors)

**Fig. 3:**
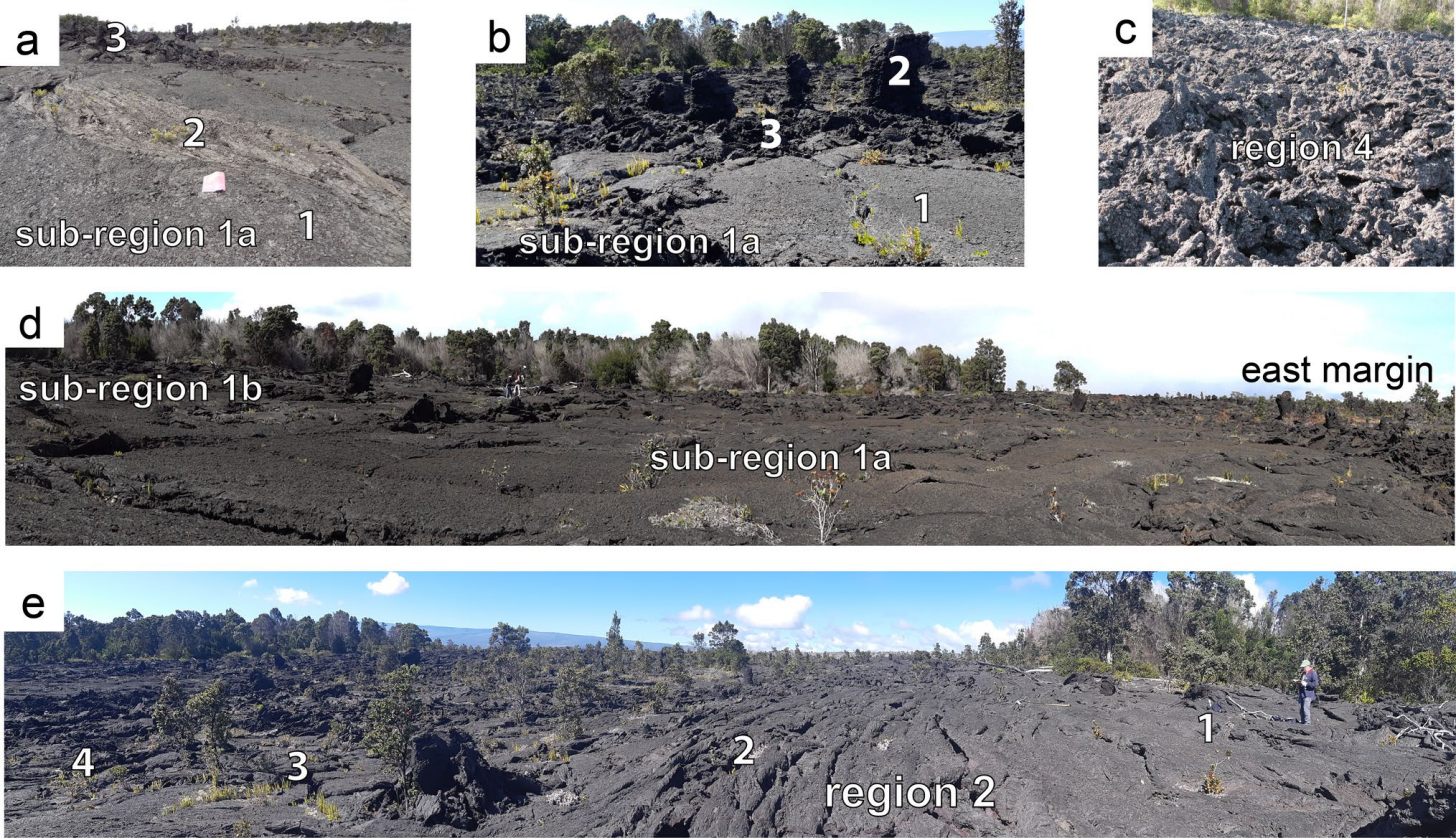
Textural features of July 1974 flow surfaces. High resolution pictures are provided in Figure S2. **a.** Three typical textures for region 1. **1.** Resurfaced lava produced by foundering of original lava crust surrounds **2.** An unsunk relic of the original ropy pāhoehoe crust. **3.** Slabby pāhoehoe in central region 1. **b. 1.** Resurfaced pāhoehoe in area lacking conspicuous tree molds. **2**. Tree mold indicates 3–4 m of drainage occurred in this area. **3**. Broken (slabby) pāhoehoe surrounds the area of large tree molds. We suggest that where lava surrounded large tree molds, drainage was accompanied by tearing of the crust attached to the tree molds producing slabs. **c.** ʻAʻā lava immediately down-channel from the region3/region 4 contact. The field of view of the closest observable slabs is ~ 2 m. **d.** Panorama northward across region 1. The elevated region to the left is the shelly pāhoehoe lava of sub-region 1b, which drained less than sub-region 1a. The center and right of the image is the smooth and ropy ponded lava of sub-region 1a. Note figure in middle distance for scale. **e.** Panorama up-channel, across the eastern flow margin of Region 2. **1.** Lateral flow margin with subdued ropy textures and very limited drainage (compare height of small tree mold immediately above numeral 1 to height of adjacent figure). **2.** Accreted margin of the main channel. **3**. Drained smooth surface toward the outer margin of the channel (contrast height of tree mold with that in region 1). **4.** Axis of the channel where late-stage drainage was responsible for development of a slabby surface texture

This study focuses on tree molds formed by the southernmost lava flow produced during the July 1974 eruption of Kīlauea on the Island of Hawaiʻi (Fig. 2, 3). The flow travelled 2.1 km from an E–W oriented fissure located southeast of Keanakāko ‘i Crater (Fig. 2b). The fissure was active on July 19 between approximately 12:30 and 16:15 Hawaii–Aleutian Standard Time (HAST; Lockwood et al. 1999) and was estimated to have erupted 3.5 × 10^6^ m^3^ of lava to the south, at a time-averaged discharge rate of ~ 195–325 m^3^/s (Lockwood et al. 1999; Soule et al. 2004). Chevrel et al. (2019) noted variable inundation recorded by tree molds, and inferred a reduction of flow velocity with time due to a combined effect of increasing viscosity and declining output rate. Preliminary results of the current study of the tree molds are given by Kosinski et al. (2012).

## Methodology

We mapped the flow in terms of geometry, surface textures and the location and specific properties of tree molds. A total of 282 tree molds were mapped using hand-held GPS with a 3-m horizontal accuracy. Field data for the tree molds and high-resolution maps described in this section are available in Online Resource 3. In addition to location, we determined three parameters for each tree mold (Fig. 1d):

1. Maximum height of the flow during emplacement (inundation height $In$), which was measured using a plumb line inserted inside the central cavity of the tree mold from the elevation of the top of the mold to the pre-eruption paleo-surface. This carries the assumption that the base of the hollow interior of the tree mold represents the paleo-surface, and that the plumb line was able to reach the base of the tree mold, without fouling on debris. This was verified by cross-comparisons between closely space clusters of tree molds.
2. The depth of any subsequent partial drainage (drainage depth $Dr$) which was the vertical distance measured between the top of the mold and the modern crust of the surrounding lava.
3. The difference $In-Dr=Th$, which is the final thickness of the flow.

We only measured tree molds identified to be complete, and plumb line measurements have a conservative associated error of ± 0.2 m. For a smaller number of tree molds, the absolute elevation of inundation was recorded with a Leica System 500 kinematic GPS with 0.2 m accuracy (Fig. 2d).

Maximum inundation height and drainage data measured from tree molds and derived final thickness were independently interpolated at a horizontal resolution of 5 m in Matlab using the *scatteredInterpolant* class and the *natural neighbor* method. Zero inundation points were manually added at regular intervals outside the flow margins to constrain interpolation by providing the boundary location. From these three surfaces, volumes of lava that either contributed to the maximum inundation, drained or constitute the final deposit were estimated at each pixel. To account for the uncertainty of both height measurements and GPS coordinates, we performed 1000 instances of the interpolation stochastically adding a random gaussian noise on inundation, drainage and final thickness values (σ = 0.2 m) and point locations (σ = 1.5 m on both x and y coordinates). Paleo-topography was estimated by subtracting the final flow thickness from a 1-m resolution airborne lidar-generated DEM (Mouginis-Mark and Garbeil 2005) resampled to match the extent and resolution of the interpolated data. The paleo-topography was in turn used to characterize the properties of the emplacement substrate (e.g., slope and topographic constraints). The inundation height map was added to the paleo-topography to estimate and contour the maximum inundation elevation above mean sea level. A 20-cm resolution orthophoto mosaic produced from digital images taken during a helicopter overflight in January 2018 and reconstructed using *Pix4D Mapper v.3.2.14* also contributed to the general interpretation of the surface features of the flow (Fig. 2c, a high-resolution version of which is available in Online Resource 1; Biass et al. 2024a). All interpolated volumes are reported as the mean ± one standard deviation estimated across 1000 interpolations.

### Use of ʻAʻā and Pāhoehoe in this study

Incandescent basaltic lava loses heat rapidly, primarily via radiation, from its upper surface to the atmosphere and forms a quenched glassy crust, which typically passes from a viscous to brittle state in a matter of minutes (Hon et al. 1994). Basaltic flows are subdivided based on whether they form and preserve a coherent upper crust (see Macdonald 1953). The nomenclature we have adopted here builds on the tripartite classificationG by Harris et al. (2017), including transitional types between pāhoehoe and ‘aʻā as introduced by Keszthelyi et al. (2000, 2004, 2006) and namely outlined by Guilbaud et al. (2005) and Duraiswami et al. (2014).

## Results

The following section summarizes field observations across the flow and volume estimates obtained from the reconstruction of the surfaces provided by the various properties inferred from tree molds measurements (Fig. 1d).

### Geometry of the Flow

We divide the flow into four regions (Fig. 2c) based on the width of the flow and the slope of its modern surface. Regions 1 and 3 are wide (~ 400 – 600 m) and essentially flat-lying. Regions 2 and 4 are narrower (~ 180 – 250 m) and elongate with steeper average down-flow slopes of 4–6°. Regions 1, 3, and 4 are divided into sub-regions, based on surface textures and/or degree of drainage from inundation height (Fig. 3).

***Region 1:*** The flow surface in the northernmost region (1) is flat-lying (average slope angle 2–4°), up to 600 m wide, and is cut by the eruptive fissure. Lava north of the fissure drained northwestward into the Keanakāko ‘i Crater (Fig. 2b). South of the fissure, region 1 forms a high-standing section of the flow that extends southward for about 500 m (Fig. 2c). It contains pāhoehoe lava, with marked ropy textures along the lateral (east and west) margins. The pre-1974 ground surface is exposed locally within the flow near the southern margin of region 1, along a conspicuous linear feature (Fig. 2c) visible on pre-eruption photography, which was mapped as a NW-facing fault scarp by Peterson (1967). This scarp was a topographic barrier and coincides with the widest part of the July 1974 flow. The eastern part of region 1 (sub-region 1a) preserves the tallest tree molds and a surface that consists of m-scale domains of alternating ropy and re-surfaced pāhoehoe (Fig. 3a). The latter often shows no surface flow textures, except where cracks feed squeeze-outs of the underlying lava. Sharp linear contacts separate the re-surfaced pāhoehoe from the adjacent domains of ropy pāhoehoe. The western part of region 1 (sub-region 1b) is higher standing and capped by shelly pāhoehoe (Fig. 3d). The lava in region 1 had an inundation volume of 4.28 ± 0.02 × 10^5^ m^3^ and has a final volume of 2.27 ± 0.02 × 10^5^ m^3^ (Table 1).

**Table 1:** Summary of volumes estimated by independent interpolation of the inundation, drainage and final thickness heights inferred from tree mold data for each region defined in Fig. 2c. The total volume considers the area outlined in Fig. 2d (i.e., including the region north of the fissure). For each region, the mean and standard deviation calculated over 1000 iterations of the interpolation are reported (see text for method details). An estimate of the final volume obtained from the subtraction of the interpolated inundation *I* and drainage *D* surfaces is also shown as well as the error obtained when comparing with the interpolated final volume. The error is calculated as (F-(I-D))/F*100

	Volume (× 10^5^ m^3^)				Error (%)
Region	Inundation (I)	Drainage (D)	Final (F)	Final (I-D)	
Total	14.88 ± 0.04	6.18 ± 0.03	8.77 ± 0.04	8.69 ± 0.02	0.8
1	4.28 ± 0.02	1.99 ± 0.02	2.27 ± 0.02	2.29 ± 0.01	−1.1
1a	2.85 ± 0.01	1.50 ± 0.01	1.33 ± 0.01	1.35 ± 0.00	−1.5
1b	1.43 ± 0.01	0.49 ± 0.01	0.94 ± 0.01	0.94 ± 0.01	0.5
2	2.70 ± 0.02	1.38 ± 0.01	1.34 ± 0.01	1.32 ± 0.01	1.9
3	4.27 ± 0.02	1.58 ± 0.02	2.71 ± 0.02	2.69 ± 0.01	2.9
3a	3.57 ± 0.02	1.56 ± 0.02	2.01 ± 0.02	2.00 ± 0.01	0.3
3b	0.70 ± 0.01	0.02 ± 0.01	0.70 ± 0.01	0.68 ± 0.01	2.5
4	2.36 ± 0.02	0.60 ± 0.01	1.78 ± 0.02	1.76 ± 0.01	2.8
4a	1.87 ± 0.02	0.58 ± 0.01	1.30 ± 0.01	1.29 ± 0.01	0.8
4b	0.49 ± 0.01	0.02 ± 0.01	0.48 ± 0.01	0.47 ± 0.00	2

***Region 2:*** The flow narrows abruptly down-flow from region 1, to become a 150 m-wide sloping channel (average slope angle > 8° dipping southward), and then widens and divides around a topographic high which, in its southernmost tip, exposes the pre-flow surface (Fig. 2c). The lava surface drops evenly down-flow along region 2 (Figs. 2d, 3e). The surface textures of the lava are heterogeneous and the channels are capped by slabby pāhoehoe, separated by areas of unbroken smooth or indistinctly ropy pāhoehoe. Where it laps on to the southernmost tip of the topographic high, the lava surface is ropy and exhibits minor drainage. The lava in region 2 has an inundation volume of 2.70 ± 0.02 × 10^5^ m^3^ and a final volume of 1.34 ± 0.01 × 10^5^ m^3^ (Table 1).

***Region 3:*** The flow widens again to 400 m, flattens compared to region 2 (average slope angle 2°), and is marked by a surface texture with alternating tongues of smooth and slabby pāhoehoe (Fig. 2c). The eastern part of region 3 (sub-region 3a) is between 250 and 300 m wide, flat-lying and connects regions 2 and 4 (Fig. 2d). The southern part of 3a constricts into a bottleneck with an abrupt transition into ʻaʻā in region 4. The western part of region 3 (sub-region 3b) is a thin, 400 m-long tongue with no drainage. The lava in region 3 has an inundation volume of 4.27 ± 0.02 × 10^5^ m^3^ and a final volume of 2.71 ± 0.02 × 10^5^ m^3^ (Table 1).

**Fig. 4:**
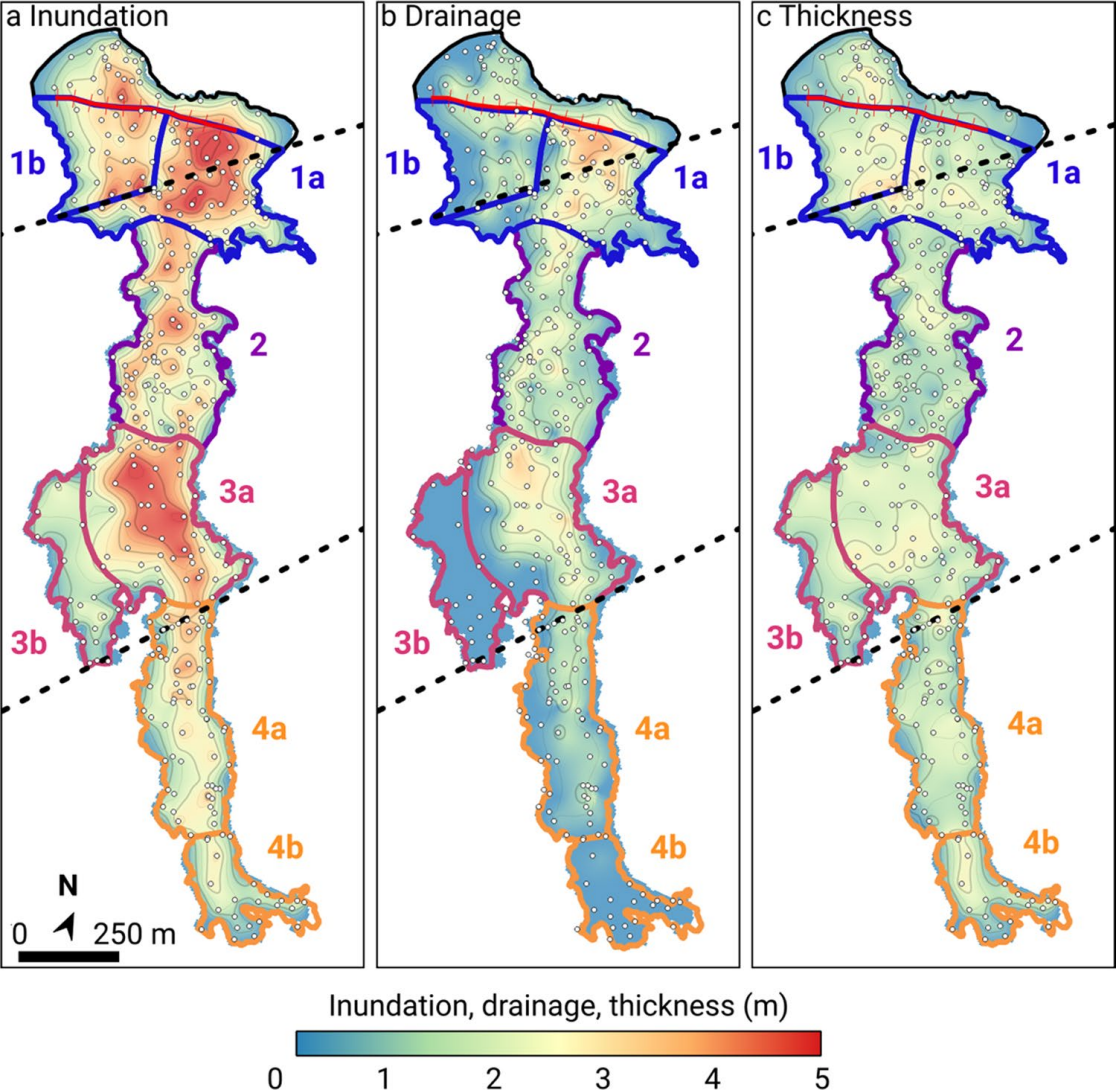
Interpolation of tree mold data showing **a.** Maximum inundation height, **b.** Drainage depth, **c.** Final flow thickness (difference between **a.** and **b.**). Contour intervals are 0.5 m. Fissure is crosshatched red line. Dashed black lines are the fault traces mapped by Peterson (1967). A high-resolution version and raw data for interpolation are provided in Figure S3

***Region 4:*** The lava in region 4 is narrower than region 3. The boundary between regions 3 and 4 coincides with a second pre-existing, NW-facing, fault (Fig. 2c) mapped by Peterson (1967). Region 4 is ~ 860 m long and ~ 180 m wide and marked by an abrupt transition to ʻaʻā morphology. The proximal part of region 4 (sub-region 4a) underwent limited drainage (1 to 2 m; Fig. 4b) with a slope of 1–2°. The distal part (sub-region 4b), with a slope of 1.5–2.5°, did not drain. The lava in region 4 has an inundation volume of 2.36 ± 0.02 × 10^5^ m^3^ and a final volume of 1.78 ± 0.02 × 10^5^ m^3^ (Table 1).

Since all estimated volumes are associated with errors of 1–2%, all following sections report only a mean value.

### Inundation heights

Sub-region 1a is generally marked by the greatest inundation, up to 5 m (Fig. 4a), except along the fault scarp mapped by Peterson (1967) where the pre-1974 ground surface is exposed at one point (Fig. 2c). The inundation surface of sub-region 1a is flat-lying, suggesting that lava ponded here at an elevation of around 1141–1144 m asl. (Fig. 2d). Sub-region 1b has lower inundation (2 to 3 m) except for a small ‘pond’ immediately north of the fault scarp. Inundation height throughout region 2 is generally 2 to 3 m and the inundation surface slopes uniformly southward from 1139 to 1120 m asl (Fig. 2d). Two small paleo-lows were inundated to heights of 3.5 to 4 m.

Sub-region 3b forms a shallow, broad area that was inundated to 1 to 2 m high (Figs. 2c and 4a). In contrast, lava inundation heights of 3.5 to 4.5 m in sub-region 3a define a flat inundation surface sloping only gradually southward from 1120 to 1116 m asl (Fig. 2d), indicating ponding. In region 4, inundation decreases smoothly down-flow from 3 m in the north to < 1 m at the toe (Figs. 2c and 4a).

### Drainage depth

In region 1, drainage depth (Fig. 4b) sharply delineates the higher western shelly pāhoehoe of sub-region 1b, with generally less than 1 m of drainage, from the resurfaced pāhoehoe of sub-region 1a, with 2 to 3.5 m of drainage. Drainage is less in region 2, typically only 2 m along the main channel and < 1 m everywhere else. Sub-region 3b has uniformly < 1 m of drainage whereas sub-region 3a has typically 2–3 m of drainage (Fig. 4b). Drainage decreases sharply in region 4; sub-region 4a has only 0.5–1 m of drainage and sub-region 4b has no drainage.

### Final thickness

**Fig. 5:**
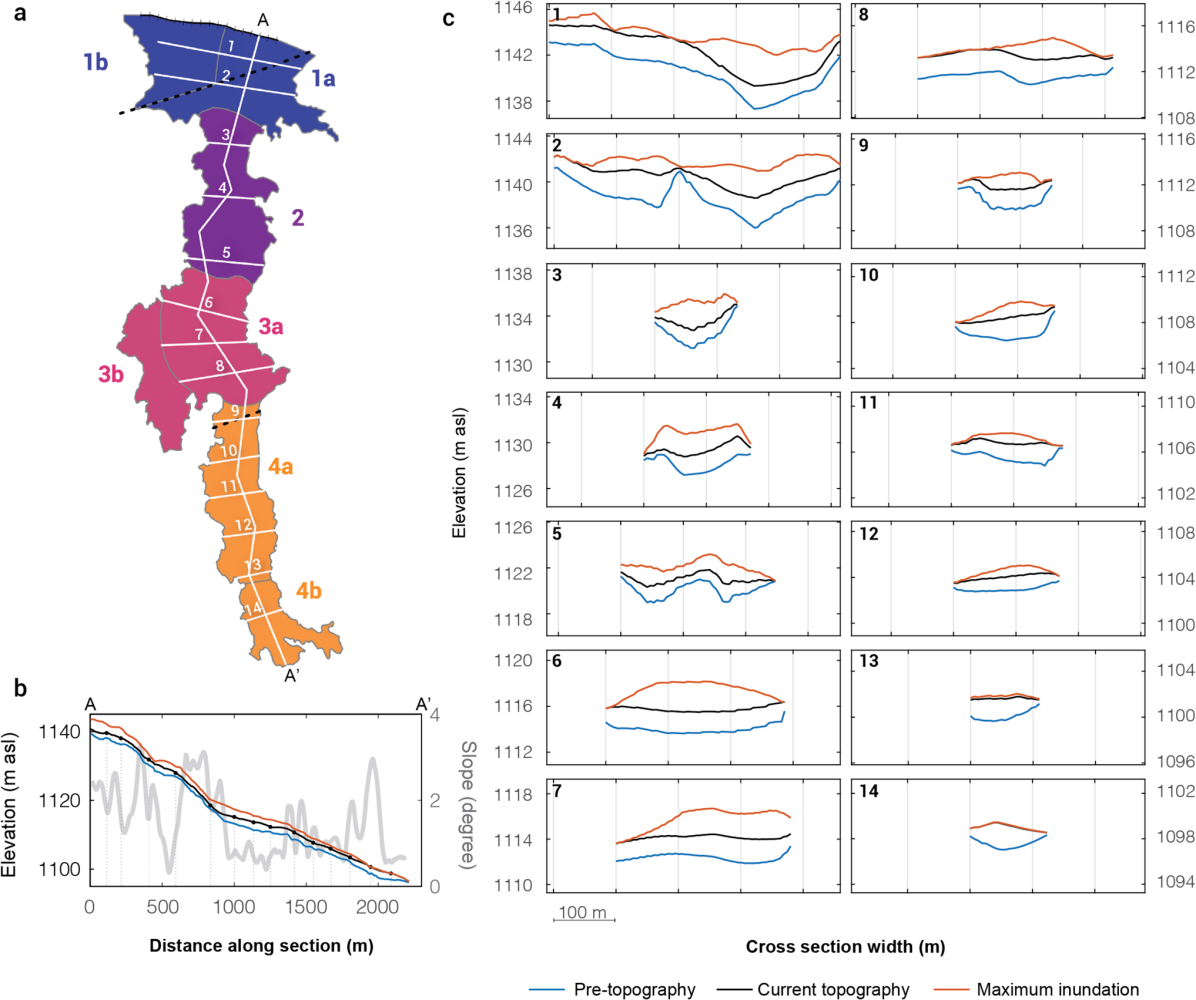
Profiles through the lava flow. **a** Flow regions defined from the geometry and surface textures of the flow and locations of the measured profiles across the flow. **b.** A-A’ profile along the flow. The location of the profile represents the line of steepest descent calculated from the inferred pre-flow topography using *TopoToolbox 2* in *Matlab* (Schwanghart and Scherler, 2014). On the left y-axis, blue is pre-eruptive surface; red is surface of maximum inundation; black is the final (modern-day) surface of the flow after drainage. On the right y-axis, grey is the associated slope smoothed with a 20 m moving mean. Vertical dotted lines show the locations of the profiles in **c**. **c**. Cross Sects. (1–14) across the flow. Blue, red and black lines as for **b**. Vertical grey grid shows 100 m interval. Note that the x and y axes are consistent across all sections

Final thickness shows little variation across the entire flow and is typically 1.5–2.5 m (Fig. 4c). The exception is a 100 m long strip in region 1, where lava remains ponded up to 3 m thick against the pre-existing fault scarp. Such thicknesses are in agreement with previously reported basaltic lava flows in Hawai’i (e.g., Katz and Cashman 2003) and elsewhere (e.g., Self et al. 2021). The final thickness data were used to calculate a bulk volume of 0.9 × 10^6^ m^3^ for the studied portion of the flow (i.e., the part to the south of the fissure, i.e., the flow area shown in Fig. 5a). For a 3.75 h fountaining duration (Lockwood et al. 1999), this corresponds to a total flow time-averaged discharge rate of ~ 67 m^3^s^−1^, substantially lower than rates inferred in previous publications (Sect. 1.1).

### Pre-eruption topography

A three-dimensional (3D) reconstruction of the pre-eruption topography is available in Online Resource 4. The slope of the topography along the axis of the flow ranges from 0.5° (region 1) to 2.5° (region 2). The pre-eruptive surface in the north of region 1 forms a broad basin-like feature, deepening to the east (Fig. 5, profile 1). To the south it is crossed obliquely by the 3-m-high fault scarp mapped by Peterson (1967) (Fig. 5, profile 2). To the northwest, lava ponded against this steep scarp (sub-region 1b). To the east is the broader pond-like feature that coincides with sub-region 1a.

The transition to region 2 is marked by an increase in pre-eruptive slope, to > 2°, that extends ~ 500 m down-flow to region 3, where the topography flattens. The pre-eruptive topography indicates that region 2 was initially a relatively steep-sided valley (Fig. 5, profiles 3 and 4). The drainage data (Fig. 4b) suggest that this is where channels draining from sub-regions 1a and 1b merged; this inference is supported by the spatial relationships between pāhoehoe textures observed in the field and visible on the high-resolution aerial image (Fig. 2c). Farther down-flow, the confining valley of region 2 divides into two (Fig. 5, profile 5) around a conspicuous N–S aligned high region in the pre-eruptive topography, which forms the kīpuka (a window where the pre-eruption surface is exposed) on the southern boundary of region 2 (Fig. 2c), to merge again southward in region 3 (compare Fig. 5, profiles 5 and 6) over a flatter region (slope ~ 0.7°). On the surface, valley thalwegs are expressed by the presence of slabby and rubbly pāhoehoe incising ropy pāhoehoe (Fig. 2c; Fig. 3e). The pool-like sub-region 3a is the continuation of outlet of the confining valley, while sub-region 3b is a thin but broad flanking sheet of lava (Fig. 5, profiles 7 and 8). Down-flow of region 3 the shallow topographic feature confining the flow narrowed and its sides steepened (Fig. 5, profiles 9 and 10) marking the start of region 4. In region 4, the valley widened (Fig. 5, profiles 11 and 12) and flattened to an average slope of ~ 1°. The end of the flow is characterized by a steeper and narrower topographic constraint, which channeled the lava into a narrow ʻaʻā tongue (Fig. 5, profiles 13 and 14).

### Emplacement process

Volume budgets inferred from Fig. 4 and summarized in Table 1 help unravel the temporal evolution of the flow emplacement between regions 1 and 3. Firstly, the volume drained from region 1 (1.99 × 10^5^ m^3^) accounts for only three-quarters of the inundation of region 2 (2.70 × 10^5^ m^3^). This indicates that a percentage of the lava that entered region 2 was dynamically transferred without contributing to the inundation height of region 1. Similarly, the drainage volumes from region 2 (1.38 × 10^5^ m^3^) or combined regions 1 and 2 (3.37 × 10^5^ m^3^) represent only a third and three-quarters of the inundation volume of region 3 (4.27 × 10^5^ m^3^), respectively. We therefore infer that up to a quarter of the total volume of region 3 (i.e., ~ 1 × 10^5^ m^3^) was directly transferred from the fissure without contributing to ponding in regions 1 and 2. Although based on a time-independent assumption, this calculation implies that over three-quarters of the volume of region 3 was emplaced at a stage during which the activity at the fissure was waning and regions 1 and 2 were undergoing drainage. The total volume of region 4 equals the volume drained from sub-region 3a (~ 1.6 × 10^5^ m^3^; Table 1). This suggests that region 4 was the result of a single episode of drainage from lava previously stored in sub-region 3a.

## Discussion

The inundation and drainage data reveal that parts of the July 1974 flow underwent periods of ponding and subsequent drainage, particularly evident in regions 1 and 3 (Fig. 4a, b). Evidence for ponding and drainage is also found in crustal textures at outcrop scale (Fig. 3a, 3b), where, resurfaced lava, produced by foundering of original lava crust (label 1 on Fig. 3a) surrounds relics of the original ropy pāhoehoe crust which did not sink (label 2 on Fig. 3a). Ponding occurs when the influx of fresh lava into a region, or sub-region, exceeds outflow. We distinguish two ponding mechanisms: *static ponding* dominantly controlled by confinement within a local topographic low, and *dynamic ponding* dominantly controlled by the balance between inflow and outflow rates. Both mechanisms appear to have operated in the July 1974 flow. In the northern part of region 1, lava ponded statically behind the topographic barrier formed by the scarp of the pre-existing fault (Fig. 2c; Fig. 4a; Fig. 5, profile 2). In the southern part of region 1a, lava ponded at the narrowing of the channel into region 2 (Fig. 5, profiles 2 and 3), indicating dynamic ponding. The boundary of regions 3 and 4 combines both a fault scarp (Fig. 2c; Fig. 4a; Fig. 5b) and a narrowing of the channel (Fig. 5, profiles 8 and 9), indicating that both static and dynamic ponding likely played a role in causing ponding in region 3a.

Ponding is therefore an important control of flow emplacement dynamics, creating temporary “reservoirs” (Orr et al. 2023), with two consequences. Firstly, the drainage of these reservoirs may have led to discharge rates that, late in the eruption, exceeded waning discharge rates at the vent (Patrick and Orr 2012). Secondly, temporary storage of lava in ponded under pāhoehoe crusts gave additional time for cooling, outgassing, and crystallization of the lava, which may have subsequently induced the transition to ʻaʻā morphology on drainage from regions 3 to 4 (e.g., Peterson and Tilling 1980). Consequently, the evidence for repeated ponding or “staging” during the emplacement of the July 1974 flow adds complications to the time-independent emplacement model. The following sections propose a new conceptual emplacement model for the July 1974 flow.

### Emplacement History

**Fig. 6:**
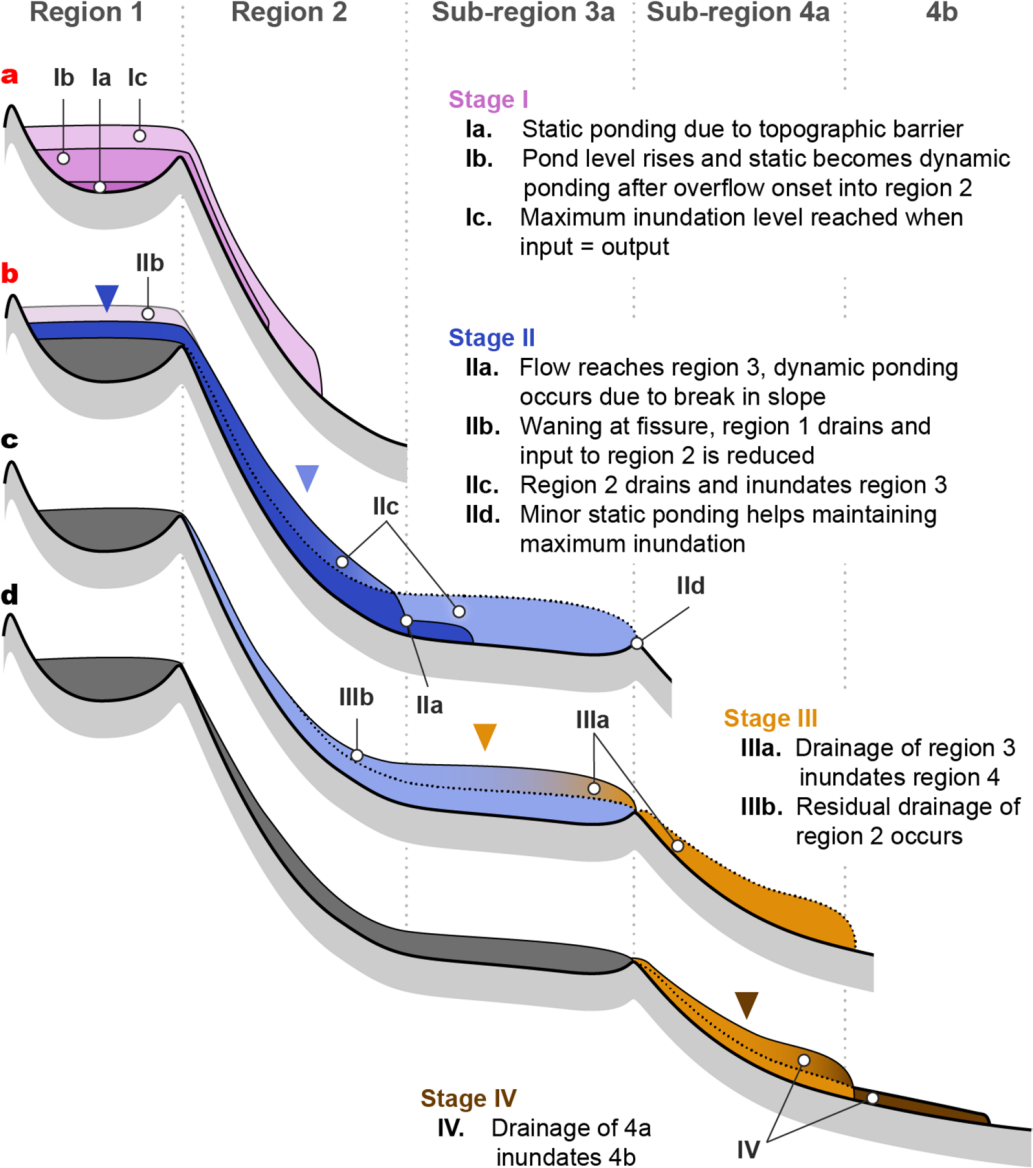
Conceptual model of the multi-stage emplacement of the southernmost part of the July 1974 flow. Regions and sub-regions correspond to those defined in Figs. 2 and 4. Triangles show foci of drainage. The red **a** and **b** labels indicate eruption stages during which the fissure is inferred to be active. The figure is not to scale

Based on the interpretation of surface flow textures, tree mold data, and 3D interpolation and volume estimates, we propose a staged and time-dependent conceptual model of flow emplacement for the July 1974 flow (Fig. 6).

### Stage I

**Fig. 7:**
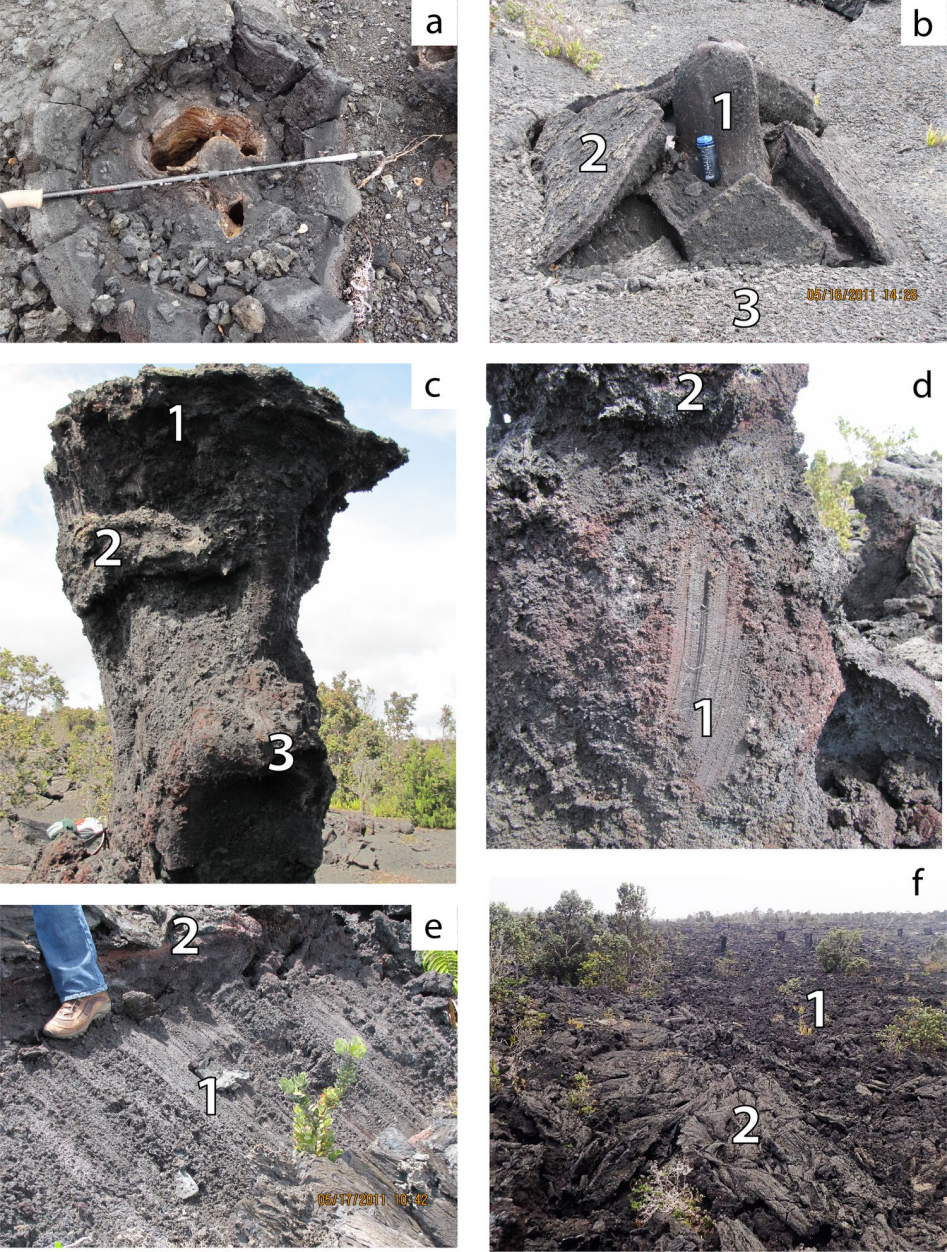
Small scale textures indicative of flow dynamics listed in inferred order of increasing flow velocity. Panel **a.** is during initial flow inundation of region 1 and **b.** through **d.** and** e**. and **f.** are features formed during subsequent drainage of regions 1 and 2 respectively. **a.** Cross section through a tree mold which initially formed around four or five delicate branches of a tree, demonstrating the gentle, slow, non-destructive emplacement of the first-arrived lava. **b.** Blind tree mold (**1**) in upper sub-region 1a with stacked crustal plates (**2**) that represent broken remnants of the crust that formed at the maximum inundation by the lava flow. Their location shows that drainage occurred from beneath the crust, which remained attached to the tree mold. The plates ultimately broke free but any subsequent drainage was too weak to raft them away from their host. (**3**) In situ crust. **c.** Multiple crustal remnants developed on a 4-m-high tree mold during staged drainage of region 1a. **(1)** Crust formed at maximum inundation. **(2)** and **(3)** are benches marking crustal level during two later standstills during drainage. **d.** Tree mold in lower sub-region 1a showing near vertical gouge marks (**1**) created as the original crust slid down the mold during rapid drainage. The orientation of the gouges suggests that the crust moved initially almost vertically before being carried away by the draining flow. Intact remnants of the crust are preserved at (**2)**. **e.** Gouges (**1**) formed during more rapid removal of crust preserved on the sloping wall of the region 2 channel, note contrast in orientation of gouges to **d**. Drainage is from left to right. In situ crust (**2**) is preserved at the level of the boot. **f.** Slabby pāhoehoe late-stage drainage channel (**1**) close to western margin of region 2 inboard of higher region (**2**) showing weakly disrupted ropy pāhoehoe formed during the initial flow into region 2

At the start of the eruption, vesicular pāhoehoe lava flowed dominantly southward from the fissure through region 1, enclosing and preserving small-scale features of “drowned” trees in the forest (Fig. 7a). Lava then inundated the adjacent higher standing sub-region 1b. The lava in sub-region 1b, generally thinner than in sub-region 1a, preserved shelly pāhoehoe fabric without convective overturn or significant drainage. In sub-region 1a lava initially ponded statically, filling low-lying, confined parts of the pre-existing topography (Fig. 7a, Online Resource 3). The flow top rose until reaching a sufficient elevation for a portion of the lava to bypass storage in region 1 and enter region 2. Inundation continued and reached a maximum depth of 5.3 m in sub-region 1a, well above the level of pre-existing relief. This observation suggests that the inundation height in sub-region 1a continued to rise after onset of inundation in region 2. This implies that the input of lava from the fissure temporarily exceeded the rate of transfer to region 2, which caused some dynamic ponding in sub-region 1a (Fig. 6a). At maximum inundation, the sub-region 1a lava underwent partial convective overturn and foundering of the crust (Fig. 3a). New crust formed and its final thickness of 4–5 cm (e.g., Fig. 7b) suggests the inundation height was maintained for 16–25 min before the onset of drainage (based on the thickness–time relationship given in Hon et al. 1994). Lava in region 2 entered a channel which progressively narrowed as material was accreted to the channel margins (Fig. 3e, location 2). Region 2 shows evidence of limited inflation and drainage (Fig. 3e, location 3).

### Stage II

Volume relationships (Table 1; Sect. 3.6) suggest that the front of the flow reached region 3 before onset of drainage in regions 1 and 2 (Fig. 6b). The lava entered a flatter topography (Fig. 2d) and formed an inflated eastern pond in sub-region 3a (Fig. 4a). Some 15% of the total volume of lava entering region 3 flowed to the west with only limited drainage (sub-region 3b). The drainage of sub-region 1a began when the influx of lava from the waning fissure became smaller than the rate of transfer to region 2 (Fig. 6b). To the north, lava drained from beneath the static crust, which subsided passively and in situ without being removed by the draining lava (Fig. 7b). In the south of region 1a, the crust tore during drainage, producing near-vertical gouges on the tree molds (Fig. 7d). The drainage of region 1 proceeded in a staged fashion leading to attachment of new crust at several levels below the maximum inundation height (Fig. 7c). Drainage of region 2 produced two late-stage channels filled by slabby pāhoehoe lava and visible on Fig. 2c. The drainage was rapid enough to drag foundering crustal slabs sub-horizontally across the channel walls (Fig. 7e). Adjacent to the topographically high kīpuka in region 2, slabby pāhoehoe lapped onto the top of primary ropy lava, confirming that the channels visible on Fig. 2c are late-stage features that formed when the southern half of region 2 was drained (Fig. 7f). The boundary between regions 2 and 3 is characterized by an intricate relationship between the slabby material originating from region 2 and the smooth surface located over the inundation maximum in region 3 (Fig. 2c; Fig. 4a). We attribute this complexity to the period when the final drainage of region 2 occurred while region 3 was reaching its maximum level of inundation. Flow dynamics of sub-region 3a were influenced by both dynamic ponding, due to a break in slope in the pre-flow topography, and static ponding above the fault scarp at the boundary between regions 3 and 4 (Fig. 2c).

### Stage III

The volume drained from sub-region 3a is comparable to the total lava volume that entered region 4 (Table 1). This suggests that region 4 is dominantly the result of a single episode of drainage out of region 3 after drainage was complete from regions 1 and 2 and the activity at the fissure had ceased (Fig. 6c). About half of region 3a shows inundations ≥ 4 m (Fig. 4a), suggesting a significant episode of ponding. We therefore argue that sub-region 3a acted as a temporary reservoir prior to transfer of material to region 4.

### Stage IV

In the final stage of the flow emplacement, a small amount of lava was initially dynamically ponded in sub-region 4a but then drained, flowing into and forming the sub-region 4b lava. We suggest that the limited supply combined with a widening of the flow in different lobes and a flattening topography (Fig. 5b) prevented any inflation or drainage. Flow emplacement ceased after the last drainage of lava out from sub-region 4a.

### What defines an eruption’s duration?

The only duration quoted for the July 1974 lava flow (3.75 h) is taken as the duration of fissure fountaining given in Lockwood et al. (1999). We suggest that here, and perhaps for other complex lava flows, the emplacement event of the lava probably out-lasted fountaining activity at the source, as a consequence of progressive downslope breaching of at least two lava ponds after supply from the fissure had ceased. A further consequence of staged ponding and drainage is that drainage fluxes occasionally exceed the time-averaged eruption flux. Both interpretations have significant implications for hazard duration for such lavas.

### What drove the abrupt transition from Pāhoehoe to ʻAʻā?

We suggest that the sharp change in texture reflects both marked decline in lava effusion rate (Chevrel et al. 2019) and a significant lava-ponding in lower region 3, before some lava drained to form region 4. Assuming region 3 acted as a temporary storage that favored crystallization-, outgassing-, and cooling-driven change of rheology in situ before drainage to sub-region 4a, the breaching of this pond could have led to a rapid outpouring from region 3 with a correspondingly high strain rate, contributing to the shift to ʻaʻā (Peterson and Tilling 1980; Kilburn 1981; Cashman et al. 1999).

## Conclusions

Lava flows are a frequent, often highly destructive hazard at many basaltic volcanoes, and risk increases sharply as the slopes of these volcanoes become densely populated. However, complete emplacement histories are based on detailed reconstruction of the history of lava flows as captured in three dimensions. The July 1974 flow from Kīlauea on the Island of Hawaiʻi has previously been described as the product of a single, continuous emplacement event (Lockwood et al. 1999), but our spatiotemporal reconstruction, based on field on field mapping of texture relationships and surface reconstruction using tree molds, reveals a more complex time-dependent flow evolution. Specifically, it identifies four staging events characterized by episodes of both statically- and dynamically-induced ponding, the latest of which having occurred after activity had ceased at the fissure. These conclusions further highlight how ponding and drainage can induce a complex, staged emplacement of basaltic lava flows, with two main consequences. Firstly, staged emplacement and ponding can extend the duration of the emplacement event and the period of hazard, possibly delaying the emplacement of a new portion of the flow from stagnant reservoirs after activity at the source has ceased (Orr et al. 2023). Secondly, ponding can reduce the previously estimated flow volume and leads to episodic drainage fluxes that must exceed the time-averaged emplacement flux (Orr et al. 2023). Overall, our results underline how careful observation and reconstruction of well-constrained eruptions (e.g., Chevrel et al. 2019; Dietterich et al. 2021; deGraffenried et al. 2021) can contribute to building accurate models of lava flow emplacement and inform forecasting of future hazardous eruptions at the most active basaltic volcanoes worldwide.

## Supplementary Information

Below is the link to the electronic supplementary material.

Supplementary file1 (PNG 29870 KB)

Supplementary file2 (PDF 142818 KB)

Supplementary file3 (PNG 5626 KB)

Supplementary file4 (FIG 1890 KB)

Supplementary file5 (DOCX 262 KB)
