# Supplementary figures and images for: Complex staged emplacement of a basaltic lava: The example of the July 1974 flow of Kīlauea
Source: Bull Volcanol. 2025 Mar 31;87(4):30. doi: 10.1007/s00445-025-01817-0 (PMC11958447; doi:10.1007/s00445-025-01817-0)

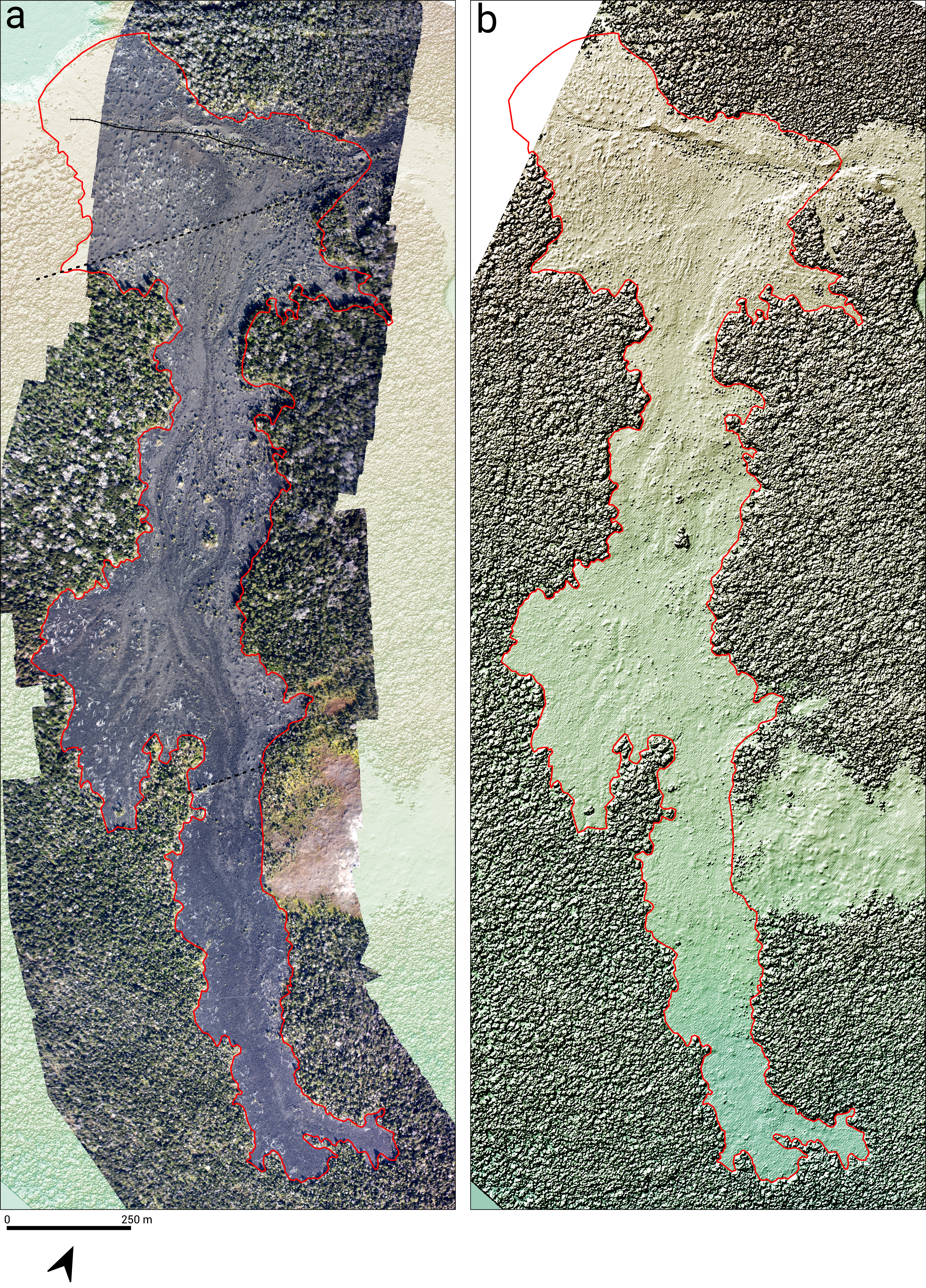

Supplement: Supplementary file 1 — Supplementary file1 (PNG 29870 KB) [file 445_2025_1817_MOESM1_ESM.png]

a

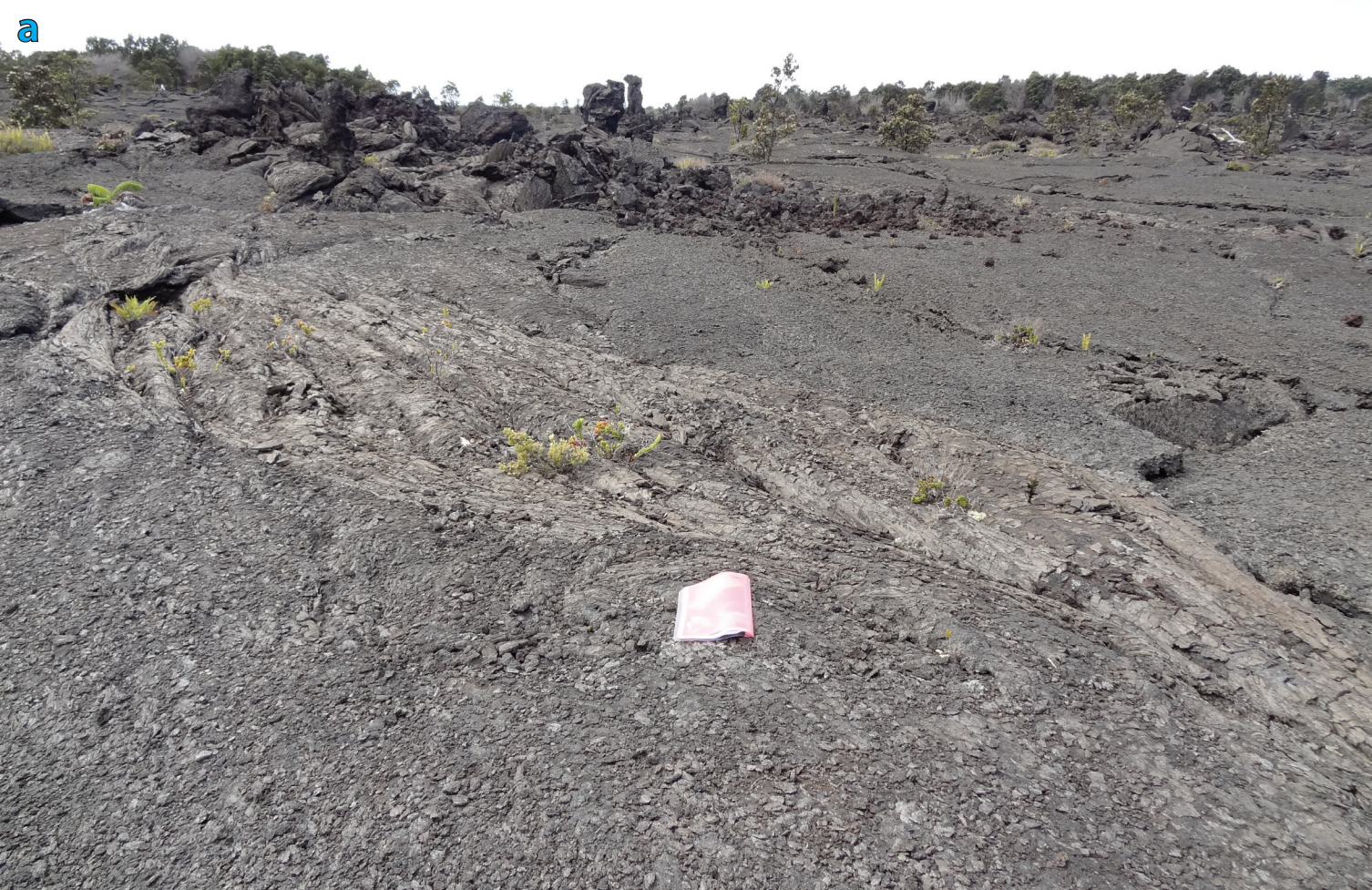

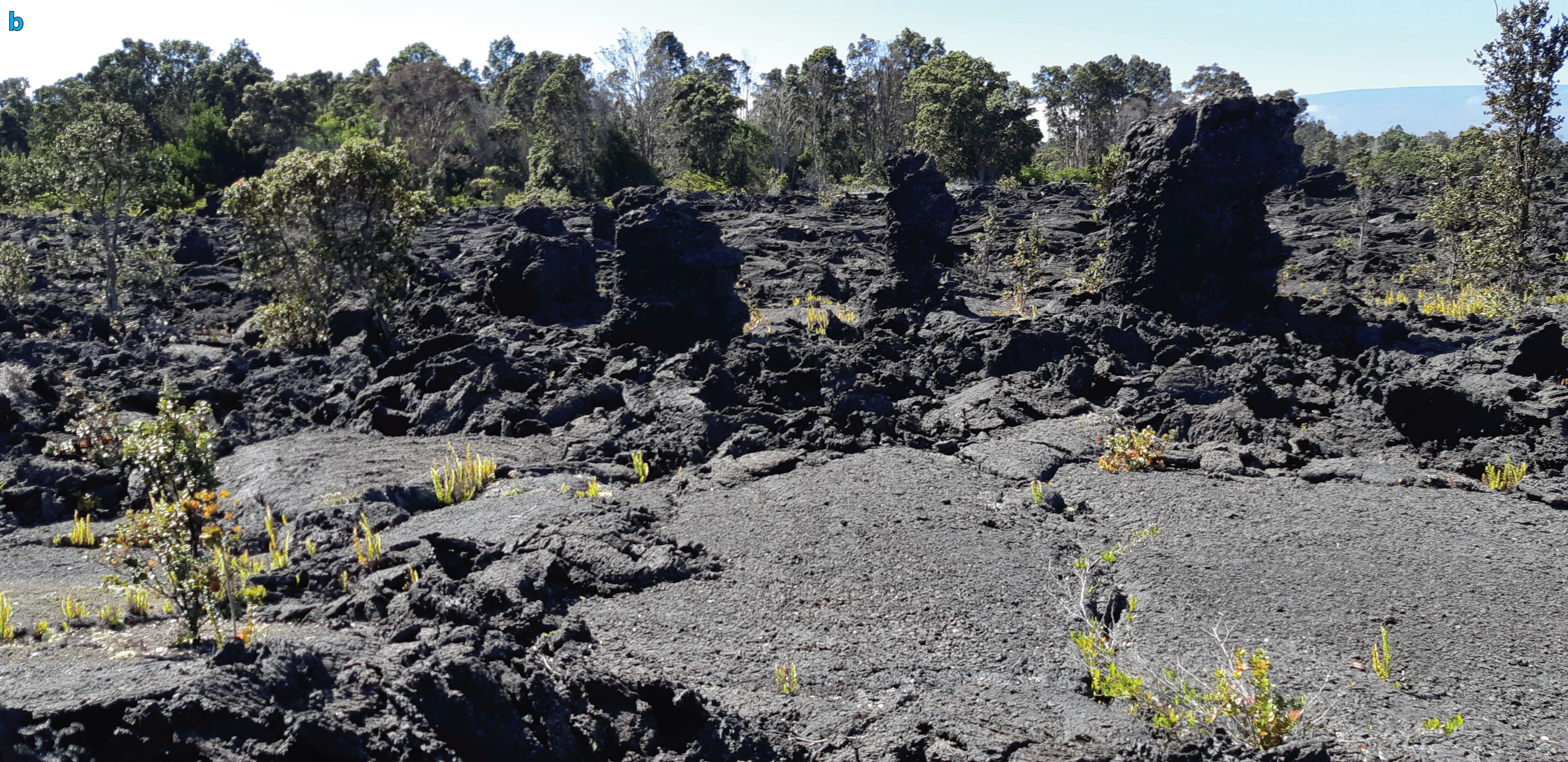

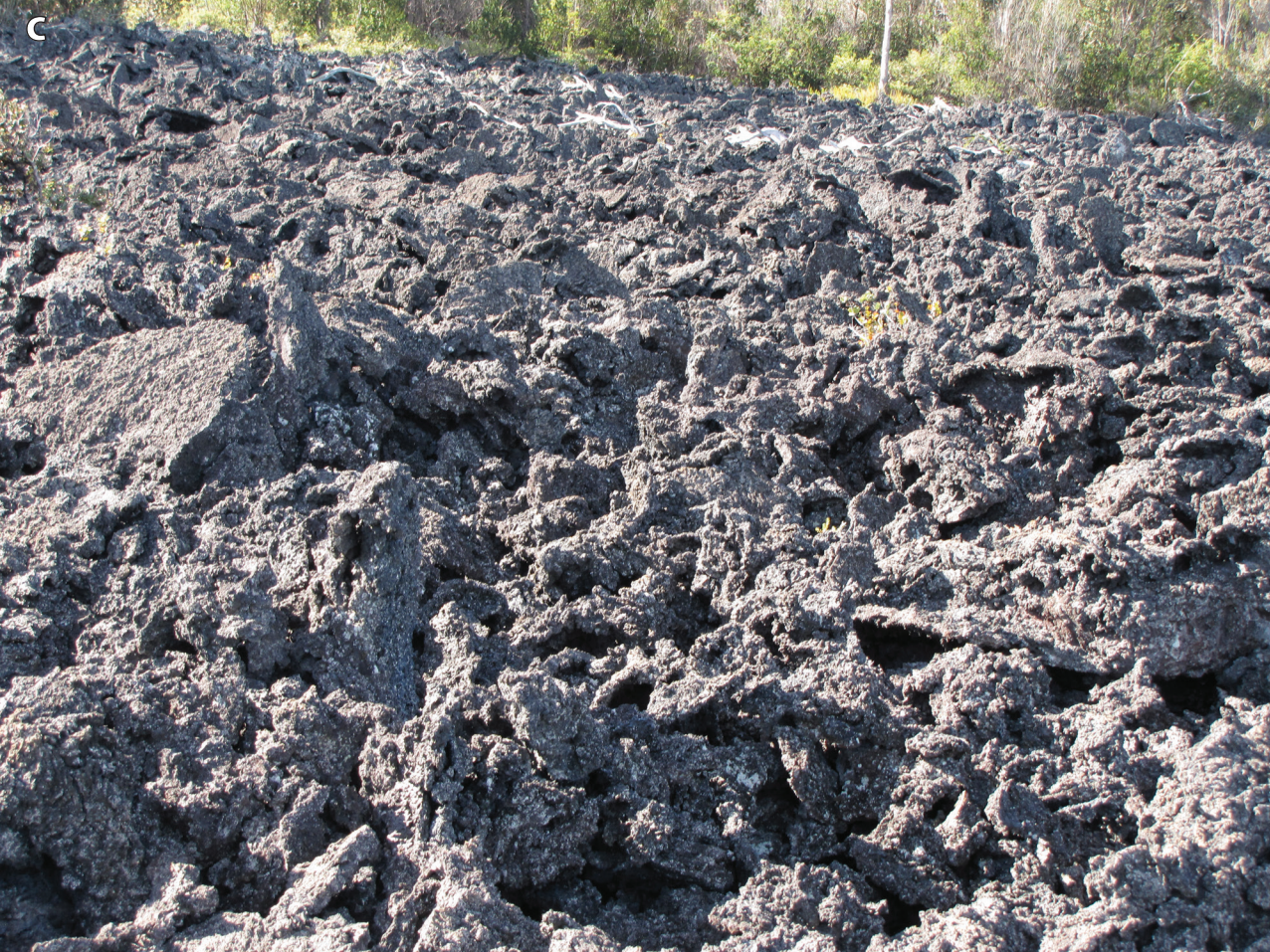

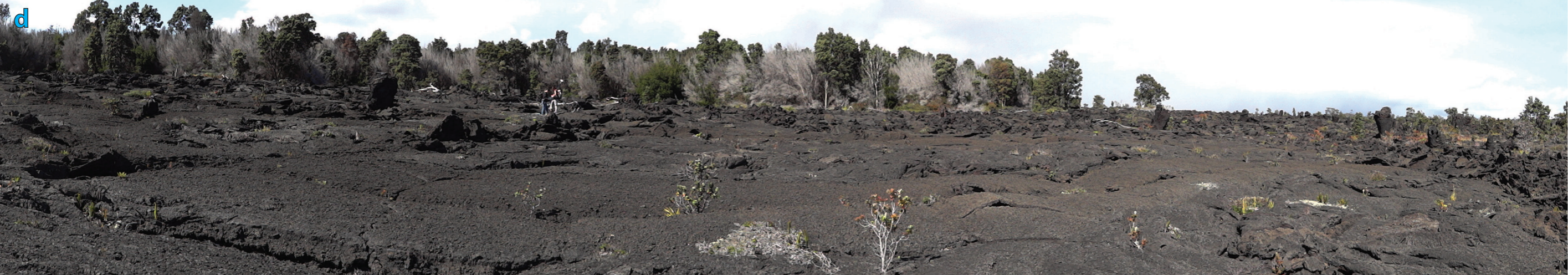

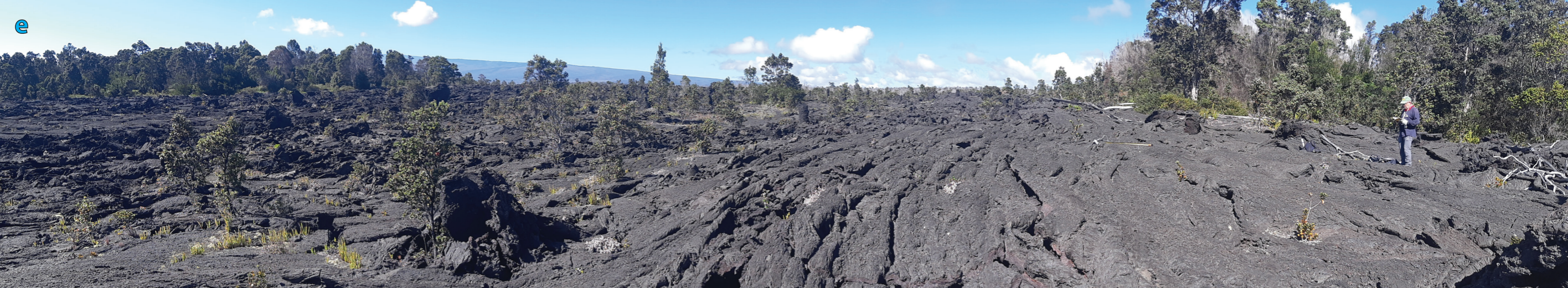

Supplement: Supplementary file 2 — Supplementary file2 (PDF 142818 KB) [file 445_2025_1817_MOESM2_ESM.pdf]

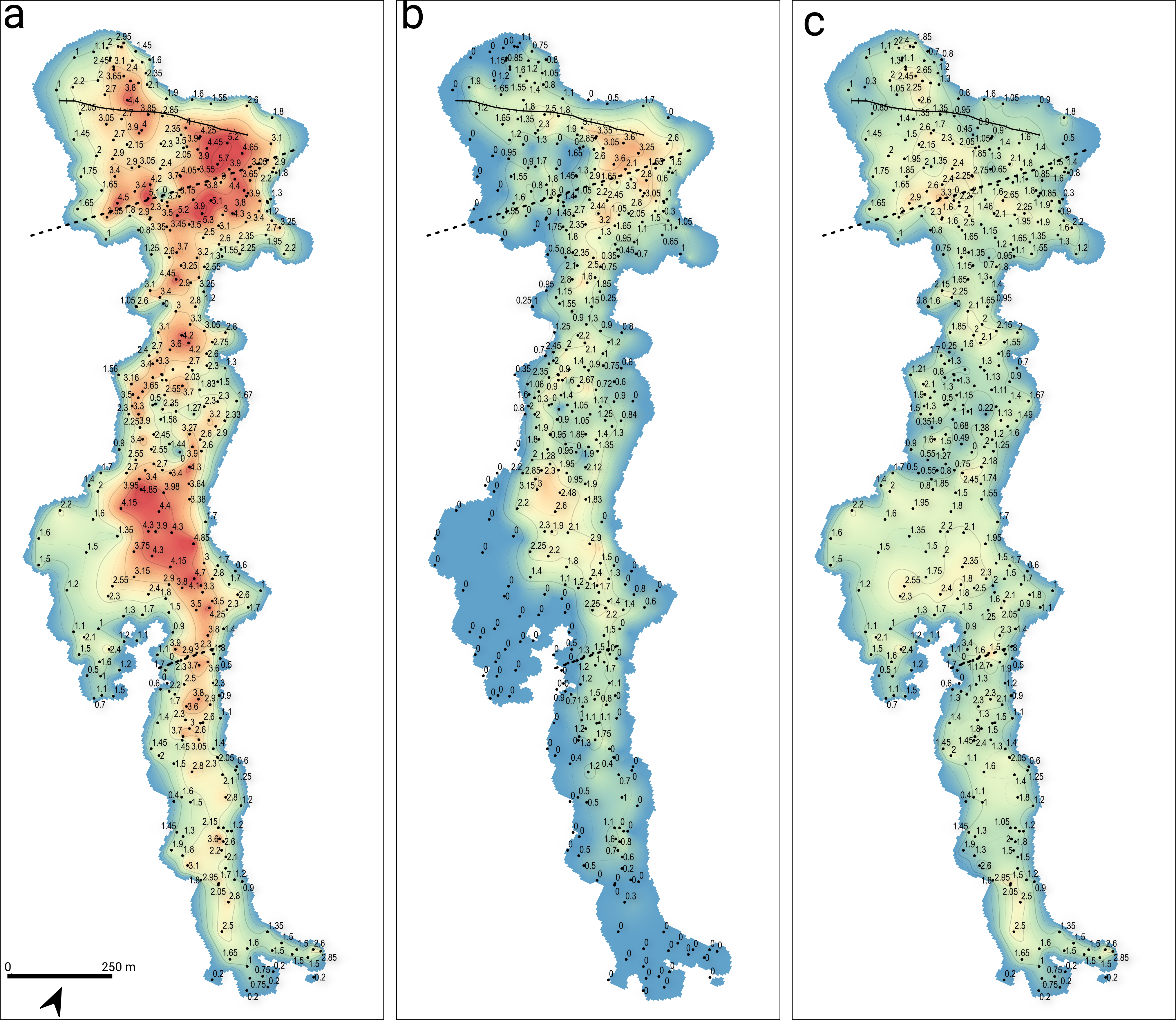

Supplement: Supplementary file 3 — Supplementary file3 (PNG 5626 KB) [file 445_2025_1817_MOESM3_ESM.png]
