# Supplementary material for: Complex staged emplacement of a basaltic lava: The example of the July 1974 flow of Kīlauea
Source: Bull Volcanol. 2025 Mar 31;87(4):30. doi: 10.1007/s00445-025-01817-0 (PMC11958447; doi:10.1007/s00445-025-01817-0)
Supplement: Supplementary file 5 — Supplementary file5 (DOCX 262 KB) [file 445_2025_1817_MOESM5_ESM.docx]

**Supplementary information to: Complex staged emplacement of a basaltic lava: The example of the July 1974 flow of Kīlauea**

**S. Biass^1,2*^, B.F. Houghton^1^, E.W. Llewellin^3^, K.C. Curran^1^, T. Thordarson^4^, T.R. Orr^5^, C.E. Parcheta^6,7^, P. Mouginis-Mark^8^**

^1^ Department of Earth Sciences, University of Hawai‘i at Mānoa, Honolulu, Hawaii 96822, USA

^2^ Department of Earth Sciences, University of Geneva, CH-1205 Geneva, Switzerland

^3^ Department of Earth Sciences, Science Labs, Durham University, Durham, DH1 3LE, UK.

^4^ Faculty of Earth Sciences, University of Iceland, 101 Reykjavík, Iceland
^5^ U.S. Geological Survey, Alaska Volcano Observatory, Anchorage, AK 99508, USA

^6^ U.S. Geological Survey, Hawaiian Volcano Observatory, Hawaii Volcanoes National Park, HI 96718, USA

^7^ University of Alaska Fairbanks, Alaska Earthquake Center, Fairbanks, AK 99775, USA

^8^ Hawai‘i Institute of Geophysics and Planetology, University of Hawai‘i at Mānoa, Honolulu, Hawaii 96822, USA

Corresponding author: Sebastien Biass (sebastien.biasse@unige.ch)

**Supplementary information**

Online Resource 1

**a.** 20-cm resolution orthophoto constructed from oblique aerial photographs taken during a helicopter overflight in January 2018 using the software *Pix4D Mapper v.3.2.14* (Biass et al. 2024). Any use of trade, firm, or product names is for descriptive purposes only and does not imply endorsement by the U.S. Government. **b.** Hillshade of the present-surface LiDAR elevation data of Mouginis-Mark and Garbeil (2005).

Online Resource 2

Original high-resolution pictures as those presented in Figure 3 of the manuscript. Refer to the manuscript for the full detailed caption.

Online Resource 3

High-resolution version of Figure 4 in the manuscript with additional raw data (m) used for interpolation. **a.** maximum inundation height, **b.** drainage depth (A2) and **c.** final flow thickness. Black dots are sampled tree molds, numbers are their associated values measured in the field and the surface is the interpolation described in the main text. Fissure is cross-hatched black line. Dashed black line are the fault scarps mapped by (Peterson 1967).

Online Resource 4

Interactive *Matlab* figure object representing the pre-flow topography obtained by subtracting the final flow thickness (Online Resource 3c) from the DEM of Mouginis-Mark and Garbeil (2005). The coloured lines represent the lines of steepest descent computed using *TopoToolbox* v2 (Schwanghart and Scherler 2014). Note that the figure can also be opened with the free alternative to *Matlab* (<https://octave.org>). A non-interactive figure is shown below.


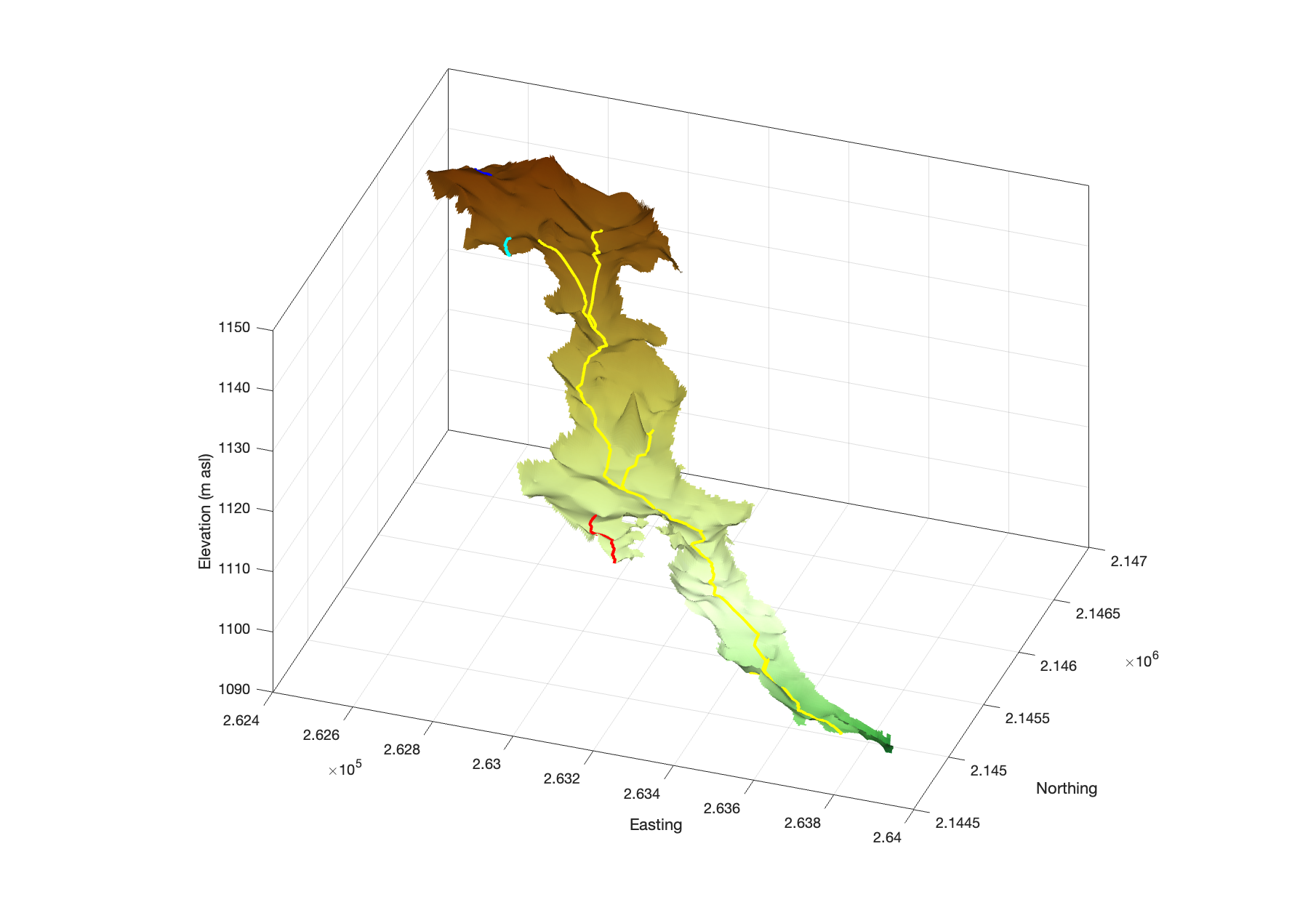


**References**

Biass S, Houghton B, Llewellin E, et al (2024) Orthophoto mosaic of the July 1974 lava flow of Kīlauea. [Data set]. Zenodo. <https://doi.org/10.5281/zenodo.11243852>

Mouginis-Mark PJ, Garbeil H (2005) Quality of TOPSAR topographic data for volcanology studies at Kilauea Volcano, Hawaii: An assessment using airborne lidar data. Remote Sensing of Environment 96:149–164. https://doi.org/10.1016/j.rse.2005.01.017

Peterson D (1967) Geologic map of the Kilauea Crater quadrangle. Hawaii: US Geological Survey Geologic Quadrangle Map GQ-667, scale 1:24000

Schwanghart W, Scherler D (2014) Short Communication: TopoToolbox 2 – MATLAB-based software for topographic analysis and modeling in Earth surface sciences. Earth Surface Dynamics 2:1–7. https://doi.org/10.5194/esurf-2-1-2014
